# Supplementary material for: Developmental 'awakening' of primary motor cortex to the sensory consequences of movement
Source: eLife. 2018 Dec 21;7:e41841. doi: 10.7554/eLife.41841 (PMC6320070; doi:10.7554/eLife.41841)
Supplement: Transparent reporting form [file elife-41841-transrepform.pdf]

1st Floor
24 Hills Road
Cambridge CB2 1JP, UK

P 01223 855340
W elifesciences.org
T @elife

eLife's transparent reporting form

We encourage authors to provide detailed information *within their submission* to facilitate the interpretation and replication of experiments. Authors can upload supporting documentation to indicate the use of appropriate reporting guidelines for health-related research (see EQUATOR Network), life science research (see the BioSharing Information Resource), or the ARRIVE guidelines for reporting work involving animal research. Where applicable, authors should refer to any relevant reporting standards documents in this form.

If you have any questions, please consult our Journal Policies and/or contact us: editorial@elifesciences.org.

Sample-size estimation

- You should state whether an appropriate sample size was computed when the study was being designed
- You should state the statistical method of sample size computation and any required assumptions
- If no explicit power analysis was used, you should describe how you decided what sample (replicate) size (number) to use

Please outline where this information can be found within the submission (e.g., sections or figure legends), or explain why this information doesn't apply to your submission:

We chose group sample sizes based on our extensive experience performing these kinds of studies in infant rats, the variability that we normally find, and the effect sizes that we expected. We recorded from a large number (at least 6 per group) of animals, recorded neural data from several (and sometimes dozens) of neurons per animal, and looked at the neural responses to dozens or even hundreds of behavioral events for each unit and for each animal.

Replicates

- You should report how often each experiment was performed
- You should include a definition of biological versus technical replication
- The data obtained should be provided and sufficient information should be provided to indicate the number of independent biological and/or technical replicates
- If you encountered any outliers, you should describe how these were handled
- Criteria for exclusion/inclusion of data should be clearly stated
- High-throughput sequence data should be uploaded before submission, with a private link for reviewers provided (these are available from both GEO and ArrayExpress)

Please outline where this information can be found within the submission (e.g., sections or figure legends), or explain why this information doesn't apply to your submission:

Several components of the present experiment replicate previous results. Further, both laminar recordings and control groups of the disinhibition experiment replicate the findings of the initial experiment. Data were only excluded if no neurophysiological data was collected or if histology showed that the recording was not performed in the correct nuclei or cortical area.

Statistical reporting

- Statistical analysis methods should be described and justified
- Raw data should be presented in figures whenever informative to do so (typically when N per group is less than 10)
- For each experiment, you should identify the statistical tests used, exact values of N, definitions of center, methods of multiple test correction, and dispersion and precision measures (e.g., mean, median, SD, SEM, confidence intervals; and, for the major substantive results, a measure of effect size (e.g., Pearson's r, Cohen's d)
- Report exact p-values wherever possible alongside the summary statistics and 95% confidence intervals. These should be reported for all key questions and not only when the p-value is less than 0.05.

Please outline where this information can be found within the submission (e.g., sections or figure legends), or explain why this information doesn't apply to your submission:

We included raw data for all individual neurons, report exact p-values whenever appropriate and provide summary statistics in the two included tables (including animal and neural N's, means, standard deviations, and when data were not normally distributed, 1st and 3rd quartiles, see Table 1 and Table 2). The significant p-value is always set at or below 0.05 and is reported in the text.

(For large datasets, or papers with a very large number of statistical tests, you may upload a single table file with tests, Ns, etc., with reference to sections in the manuscript.)

Group allocation

- Indicate how samples were allocated into experimental groups (in the case of clinical studies, please specify allocation to treatment method); if randomization was used, please also state if restricted randomization was applied
- Indicate if masking was used during group allocation, data collection and/or data analysis

Please outline where this information can be found within the submission (e.g., sections or figure legends), or explain why this information doesn't apply to your submission:

Pups were randomly selected from litters and, when appropriate, were randomly assigned to experimental or control groups. This information is found in the methods.

Additional data files ("source data")

- We encourage you to upload relevant additional data files, such as numerical data that are represented as a graph in a figure, or as a summary table
- Where provided, these should be in the most useful format, and they can be uploaded as "Source data" files linked to a main figure or table
- Include model definition files including the full list of parameters used
- Include code used for data analysis (e.g., R, MatLab)
- Avoid stating that data files are "available upon request"

Please indicate the figures or tables for which source data files have been provided:

eLife Sciences Publications, Ltd is a limited liability non-profit non-stock corporation incorporated in the State of Delaware, USA, with company number 5030732, and is registered in the UK with company number FC030576 and branch number BR015634 at the address 1st Floor, 24 Hills Road, Cambridge CB2 1JP | August 2014

Whenever possible, individual data points are represented within a figure, and are summarized in the included tables. Upon publication, we will upload our raw data timeseries (neural firing timecodes and behavioral event timecodes) for all included animals to Dryad.

We will upload custom MATLAB scripts for generating and fitting perievent histograms to twitch and wake movement models to github upon publication.
